# Psychometric properties of the Spanish version of the hospital anxiety and depression scale in cancer patients

**DOI:** 10.3389/fpsyg.2024.1497946

**Published:** 2025-01-21

**Authors:** Ana L. Vilela-Estrada, David Villarreal-Zegarra, Anthony Copez-Lonzoy, Loida Esenarro-Valencia, José C. Sánchez-Ramírez, Fernando Lamas-Delgado, Juan Ambrosio-Melgarejo, C. Mahony Reategui-Rivera, Joseph Finkelstein

**Affiliations:** ^1^CRONICAS Center of Excellence in Chronic Diseases, Universidad Peruana Cayetano Heredia, Lima, Peru; ^2^Instituto Peruano de Orientación Psicológica, Lima, Peru; ^3^Department of Biomedical Informatics, School of Medicine, University of Utah, Salt Lake City, UT, United States; ^4^Unidad de Investigación en Bibliometría, Universidad San Ignacio de Loyola, Lima, Peru; ^5^Instituto Nacional de Enfermedades Neoplásicas, Lima, Peru; ^6^Escuela de Posgrado, Universidad Peruana Unión, Lima, Peru; ^7^Instituto de Neuropsicología y Demencias, Lima, Peru; ^8^Universidad de Lima, Lima, Peru; ^9^Facultad de Ciencias de la Salud, Universidad SanIgnacio de Loyola, Lima, Peru; ^10^National Institute of Health (Peru), Lima, Peru

**Keywords:** depression, anxiety, cancer, psychometrics, Peru, hospitals

## Abstract

**Background:**

Although the Hospital Anxiety and Depression Scale (HADS) has been widely studied across various populations, there is still no consensus on its factor structure. This study aims to evaluate the psychometric properties of the HADS in cancer patients.

**Methods:**

Our study was cross-sectional and non-probabilistic. It involved 467 cancer patients aged 18 years and over, who were treated at a public institution specialized in oncology. The Hospital Anxiety and Depression Scale, the Beck Anxiety Inventory, and the Beck Depression Inventory were used. We evaluated their internal structure, measurement invariance, relationship with other variables, and reliability.

**Results:**

It was found that the HADS is best suited to a bifactorial structure where there is one general factor (emotional distress) and two specific factors (anxiety and depression). The HADS demonstrates invariance with respect to sex and years of education. It shows a moderate correlation with the Beck Anxiety Inventory and the Beck Depression Inventory. In addition, it presents acceptable levels of reliability and relationship with instruments used in the diagnosis of anxiety and depression.

**Conclusion:**

The HADS is best suited to a bifactorial structure in cancer populations, with comparisons across both sexes and varying levels of education. Its brevity, versatility, hospital-focused design, and extensive validation make the HADS a very important instrument in the detection of anxiety and depression in cancer patients.

## Introduction

According to the Global Burden of Disease Study 2019, anxiety and depression were the leading causes of global disability-adjusted life years (DALYs) and years lived with disability (YLDs) in mental health (GBD 2019 Mental Disorders Collaborators, 2022). Furthermore, it is estimated that anxiety disorders accounted for 28.68 million DALYs (Xiong et al., 2022) and depressive disorders contributed 49.4 million DALYs (GBD 2019 Mental Disorders Collaborators, 2022). In cancer patients, the prevalence of these disorders is higher, with anxiety affecting 9.8–10.3% and depression affecting 16.3–16.5% of patients (Mitchell et al., 2011).

Validated scales are widely used as cost-effective tools for assessing affective disorders (Ehlers et al., 2018; Siu et al., 2016). The literature highlights various instruments used to assess these affective disorders in cancer patients: the Beck Anxiety Inventory (BAI), the Beck Depression Inventory (BDI), and the Hospital Anxiety and Depression Scale (HADS) (Howell et al., 2015). Among these, the HADS is frequently cited in systematic reviews as one of the most widely used instruments for detecting affective disorders in cancer patients (Maters et al., 2013; Vodermaier and Millman, 2011). This instrument, designed for hospital populations, can be used to assess emotional distress from a psychosocial perspective (one-dimensional model) or from a clinical perspective (two-dimensional model of anxiety and depression) (Norton et al., 2013; Zigmond and Snaith, 1983).

Since its inception, the HADS has undergone extensive testing to verify both its validity and reliability in English and other languages (Al Aseri et al., 2015; Christensen et al., 2020; Lin et al., 2017; Reda, 2011; Yang et al., 2019), yielding satisfactory results in various hospital populations such as patients with heart disease, cancer, HIV, and psychiatric disorders. However, studies on the factor structure of the scale have given heterogeneous results (Norton et al., 2013). These studies used methods such as exploratory factor analysis (EFA), confirmatory factor analysis (CFA), and item response theory (IRT). These identified structural models based on one dimension (Waqas et al., 2019), two dimensions (Zigmond and Snaith, 1983), and three dimensions (Caci et al., 2003; Dunbar et al., 2000; Friedman et al., 2001). Therefore, the factorial structure of the HADS is not conclusive, which could affect the validity of the instrument.

Beyond structural analysis, measurement invariance is another essential property to consider, as it enables meaningful comparisons between groups. If measurement invariance holds between two groups, it suggests that both groups interpret and understand the construct being measured in a similar way (Putnick and Bornstein, 2016). Regarding sex, different studies support that invariance is met between men and women (Annunziata et al., 2011; Hunt-Shanks et al., 2010; Iani et al., 2014; Stott et al., 2017a; Stott et al., 2017b; Yang et al., 2019). However, the results of the analysis of invariance between different age groups have shown contradictory findings (Iani et al., 2014; Stott et al., 2017a; Stott et al., 2017b). Preliminary evidence supports invariance among patients with HIV, regardless of infection status (Yang et al., 2019), and among patients with different stages of cancer (Annunziata et al., 2011).

Convergent validity is another important property to analyze, as it refers to the strong and direct relationship expected between instruments that measure the same construct. In that sense, several studies have found a relationship between the HADS and other variables in different settings (palliative care, brain tumor, and specific clinical groups). In terms of the total HADS score, a strong and positive correlation is reported with emotional distress (Emotional Distress Detection Scale—DEDS) (Limonero et al., 2012), post-traumatic stress and demoralization (Belar et al., 2019; Mystakidou et al., 2007a), and with Psychosocial and Spiritual Needs of the Sick at the End of Life (ENP-E, in palliative patients) (Mystakidou et al., 2007b). For the anxiety subscale, a strong and positive correlation was reported with the State–Trait Anxiety Inventory (STAI), the Prostate Cancer Memorial Anxiety Scale (MAX-PC), and the DEDS subscale (Limonero et al., 2012; Mystakidou et al., 2009; Touzani et al., 2019). It showed a moderate correlation with fear of recurrence (FoR) (Hinz et al., 2015; Humphris et al., 2018; Shin et al., 2017) and a strong correlation with non-psychological variables, such as cancer-related fatigue (Fillion et al., 2003). On the depression subscale of the HADS, a strong and moderate positive correlation was reported using the Beck Depression Inventory and the Patient Health Questionnaire (PHQ-9), respectively (Mystakidou et al., 2007a; Rooney et al., 2012), while with non-psychological variables, such as cancer-related fatigue, it also showed a strong correlation (Fillion et al., 2003).

However, there is limited evidence regarding the validity of the Spanish version of the Hospital Anxiety and Depression Scale (HADS) specifically in cancer patients. Previous studies have primarily assessed the validity of the HADS in populations with other clinical conditions, such as pulmonary, rheumatological, infectious, or cardiovascular diseases (Herrero et al., 2003; Luciano et al., 2014; Quintana et al., 2003; Suárez-Mendoza et al., 2019). Importantly, research on other questionnaires has demonstrated that the validity and reliability metrics of psychometric instruments can vary significantly depending on the clinical conditions of the studied populations. Differences have been observed across general populations, individuals with endocrine disorders such as diabetes, and patients with cancer (Hinz et al., 2016; van Dijk et al., 2018). A study conducted in Chile evaluated the validity of the Spanish version of the HADS in a cancer population, but it focused exclusively on a one-factor structure related to emotional distress symptoms (Villoria and Lara, 2018). This approach may have overlooked other potential factor structures, which could provide a more nuanced understanding of the instrument’s performance. Therefore, it is crucial to explore the psychometric properties of the HADS further, considering multiple factor structures to assess its robustness and applicability in cancer patients.

Given the necessity of valid and reliable instruments for detecting affective disorders in Spanish-speaking hospital populations and considering the HADS as one of the most utilized tools for this purpose, this study aimed to evaluate the evidence of validity and reliability of the Spanish version of the HADS in cancer patients.

## Materials and methods

### Study design

Our study employs a cross-sectional design.

### Setting

The evaluation was conducted by psychologists or educational psychologists at a Peruvian public cancer institution in Lima, Peru. The hospital where the study was carried out is a highly specialized facility that receives oncology patient referrals from various regions of Peru. The evaluators were trained in administering the psychometric tests specific to the study. The scales were administered individually to oncology patients with a definitive diagnosis.

### Participants

The sample included 500 participants, who fulfilled the following inclusion criteria: being cancer patients of the National Institute of Neoplastic Diseases, are over 18 years old, and can read and write. Furthermore, participants should not present physical discomfort during the administration, nor have cognitive disabilities that limit their understanding or ability to complete the instruments of the current study. The sampling was intentionally non-probabilistic.

### Procedures and ethics

The protocol was approved by the INEN Research Ethics Committee and the Research Review Committee (N°239-2018-CIE/INEN). Participants were invited to take part in the research in accordance with conventional ethical standards. After providing written informed consent, they were given a questionnaire that included socio-demographic questions, the Peruvian adaptation of the HADS, the BDI-II, and the BAI.

### Instruments

#### The hospital anxiety and depression scale (HADS)

The HADS is a 14-item questionnaire created by Zigmond and Snaith in 1983 to measure symptoms of anxiety and depression in patients with somatic illnesses (Zigmond and Snaith, 1983). It has questions to detect cognitive symptoms of anxiety and depression. Furthermore, both subscales would provide an overall score for emotional distress (Norton et al., 2013). The scale is Likert-type, where 0 is the lowest score and 3 is the highest score to measure the symptoms experienced during the last week. The score range for the global dimension is 0–42, and the range for each subdimension of anxiety and depression is 0–21.

The HADS translation (Muñiz et al., 2013) from English to Spanish was evaluated by two independent consultants, who based their work on the original Spanish translation of the test by Zigmond and Snaith (1983). Additionally, a reverse translation (Spanish–English) was conducted for further evaluation. Finally, an analysis regarding its clarity, relevance, and appropriateness was carried out by 10 expert judges (eight psychologists and two psychometrists).

#### The Beck depression inventory—second edition (BDI-II)

The BDI-II is a 21-item multiple-choice self-report inventory created by Beck, Steer, and Brown in 1996 to measure the severity of depressive symptoms in psychiatric patients and in normal adolescents and adults (Beck et al., 1996). It has high internal consistency (*α* = 0.91) (Brenlla et al., 2013) and test–retest reliability (*α* = 0.90). The evidence of convergent validity was robust and showed strong correlations with the MMPI (*r* = 0.58) and Depression Scale of SCL-90 (*r* = 0.81). The factorial validity obtained two factors: somatic-affective and cognitive-affective, respectively.

#### The Beck anxiety inventory (BAI)

The BAI is a 21-item self-applied scale created by Beck, Epstein, Brown, and Steer in 1988 to measure the severity of anxiety symptoms in adults and adolescents (Beck et al., 1988) in psychiatric populations. It shows a high internal consistency (*α* = 0.92) and test–retest reliability over 1 week (*r* = 75) (Beck et al., 1988). In addition, the BDI has previously been validated in Spanish for a global dimension (Vizioli and Pagano, 2020).

### Statistical analysis

Five groups of analyses were conducted. First, the characteristics of the participants were evaluated (socio-demographic characteristics) and item characteristics (standard deviation and mean). Second, a confirmatory factor analysis (CFA) was used with the goal of evaluating 10 models proposed for the Hospital Anxiety and Depression Scale (Norton et al., 2013). Third, the measurement invariance was evaluated to know whether the models were adequate according to groups (sex and education levels). Fourth, the relationship was evaluated with other variables: the Beck Depression Inventory (BDI) and the Beck Anxiety Inventory (BAI). Fifth, internal consistency was evaluated with alpha and omega coefficients.

### Confirmatory factor analysis

For the analysis of the factor structure of HADS, the 10 models analyzed in a meta-confirmatory factor analysis were used (Norton et al., 2013; Zigmond and Snaith, 1983). Of these models, one was a one-dimensional model, two were correlated factor models (anxiety-depression), five were 3-factor models (four correlated factor models and one higher-order model), and two were bifactor models (one model with two orthogonal factors and one with three orthogonal factors) (Norton et al., 2013).

CFA is a statistical procedure, which allows checking of the validity of the instrument’s internal structure (Batista-Foguet et al., 2004). In this study, the CFA was used to analyze 10 models that have previously been shown to have adequate goodness-of-fit indices. In addition, for ordinal data, the weighted least squares with mean and variance adjusted (WLSMV) estimator was chosen for the CFA, and a polychoric matrix was used, as these methods are specifically designed for ordinal data (Dominguez-Lara, 2014; Li, 2016). The analysis was conducted in three stages. First, to evaluate the model’s adjustment index, the CFI, TLI, RMSEA, SRMR, and confidence interval (CI) with 90% were considered. Second, to evaluate overlapping factors, the latent correlations between dimensions were to be considered. Third, to evaluate the relevance of a general factor in bifactor models (models 9 and 10), the following indices were used: hierarchical omega (ωH), percentage of uncontaminated correlations (PUC), and explained common variance (ECV). The data would be in favor of the general factor if values of ωH ≥ 0.70, PUC ≥ 0.70, and ECV ≥ 0.60 are found (Dominguez-Lara and Rodriguez, 2017).

### Measurement invariance

A measurement invariance analysis was conducted with the aim of evaluating whether the different groups assessed, such as men and women, understand the evaluated construct equivalently (Putnick and Bornstein, 2016). Of the 10 initial models, the most parsimonious and best-fitting models (CFA) were taken. These models underwent measurement invariance analysis. The evaluation of levels of measurement invariance was carried out in two stages. In the first one, it was evaluated at the configuration and metric level; for the configuration level, the factorial structures were evaluated to be equal; for the metric level, the factorial loads were restricted to be equivalent. In the second stage, it was evaluated at the scalar level, where the intercepts were restricted to be equivalent. In both stages, the level of invariance was accepted if the variations in the CFI < 0.01. In addition, the values obtained through the DIF test were reported.

### Relationship with other variables

This study examines the relationships between the Hospital Anxiety and Depression Scale (HADS) and other established measures of depression and anxiety, specifically the Beck Depression Inventory (BDI) and the Beck Anxiety Inventory (BAI). It is anticipated that the overall HADS factor will exhibit moderate correlations with both the overall BAI and BDI scores. Furthermore, a high correlation is expected between the first HADS-specific factor, which assesses depressive symptoms, and the general factor of the BDI. Similarly, a strong correlation is anticipated between the second HADS-specific factor, which assesses anxiety, and the general factor of BAI. Specifically, we expect a stronger relationship between instruments measuring the same constructs than between those measuring different constructs. Correlation strengths are categorized as high (*r* > 0.7), moderate (*r* > 0.5), and low (*r* > 0.3) (Mukaka, 2012).

### Internal consistency analysis

To identify the consistency measure of the construct, this study performed an internal consistency analysis. Alpha and omega coefficients were used to evaluate internal consistency. In addition, they were acceptable values when the coefficients had values greater than 0.70 (Campo-Arias and Oviedo, 2008).

### Software used

R and STATA were used for the analysis. For analysis with R, the following packages were used: ‘lavaan’, ‘semTools’, ‘psych’, and ‘survey’.

## Results

### Characteristics of the participants

Initially, 500 participants were evaluated. However, 25 (5%) were excluded due to missing data on the HADS, and 8 (1.6%) were excluded for being foreigners (*n* = 8, 1.6%). The study included 467 participants. The majority of participants were female (75.6%), with ages ranging from 17 to 84 years (mean = 45.9; SD = 14.4). The majority were married or cohabiting (48.4%) and unemployed (78.4%), predominantly homemakers. The detailed characteristics of the participants are presented in Table 1.

**Table 1:** Characteristics of the participants (*n* = 467).

		*N*	%
Sex	Men	114	24.4%
	Women	353	75.6%
Age	17–19	13	2.8%
	20–29	59	12.6%
	30–39	84	18.0%
	40–49	109	23.3%
	50–59	113	24.2%
	60 to more	89	19.1%
Type of care	Outpatient clinic	185	39.6%
	Outpatient	154	33.0%
	Hospitalization	128	27.4%
Civil status	Married or Cohabiting	226	48.4%
	Divorced or Separated	52	11.1%
	Single	167	35.8%
	Widowed	22	4.7%
Educational years	At least 6 years old	79	17.0%
	7–11 years	215	46.0%
	12 to more	173	37.0%
Laboral status	Unemployed	366	78.4%
	Employee	33	7.1%
	Independent	68	14.6%
Previous psychological assistance	No	287	61.5%
	Yes	180	38.5%

### Confirmatory factor analysis

The factor analysis revealed that the one-dimensional model exhibited low goodness-of-fit indices, with CFI and TLI values below the threshold expected for a clinical instrument (< 0.95; Model 1). Consequently, this model was not considered for further analyses. Additionally, the bifactor model of the HADS with three orthogonal factors failed to converge (Model 10), leading to its exclusion. The other models had adequate goodness-of-fit indices (see Table 2).

**Table 2:** Goodness-of-fit indices and latent correlations of each of the models evaluated for HADS.

	*X* ^2^	df	CFI	TLI	RMSEA [CI 90%]	SRMR	ΦAnx-Dep	ΦRAN-Anx	ΦRAN-Dep
1. Razavi	306.3	77	0.933	0.921	0.080 [0.071–0.089]	0.064	-	-	-
2. Zigmond & Snaith	204.2	76	0.963	0.955	0.060 [0.050–0.070]	0.052	0.807	-	-
3. Moorey	191.8	76	0.966	0.960	0.057 [0.047–0.067]	0.051	0.794	-	-
4. Friedman	177.1	73	0.970	0.962	0.055 [0.045–0.066]	0.049	0.748 ^a^	0.997 ^a^	0.812 ^a^
5. Caci	212.6	74	0.960	0.950	0.063 [0.054–0.073]	0.054	0.776 ^r^	0.920 ^r^	0.965 ^r^
6. Brandberg	225.6	74	0.956	0.946	0.066 [0.057–0.076]	0.056	0.762 ^r^	0.940 ^r^	0.926 ^r^
7. Dunbar	189.0	74	0.966	0.959	0.058 [0.048–0.068]	0.050	0.714 ^n^	0.916 ^n^	0.841 ^n^
8. Dunbar, higher-order	190.3	75	0.966	0.956	0.057 [0.047–0.068]	0.050	-	0.888 ^n^	0.820 ^n^
9. Bifactor, 2 group-factors	**141.0**	**63**	**0.977**	**0.967**	**0.052 [0.040–0.063]**	**0.042**	**-**	**-**	**-**
10. Bifactor, 3 group factors					Not converging	-	-	-	-

Anx, anxiety; Dep, depression; RAN, restlessness/agitation/negative affection; ^r^, restlessness; ^a^, agitation; ^n^, negative affection; Φ, latent correlation between dimensions; *X*^2^, chi-squared; df, degrees of freedom; CFI, comparative fit index; TLI, Tucker–Lewis index; RMSEA, root mean square error of approximation; SRMR, standardized root mean square residual. Bold values mean the best model.

When analyzing the two-factor model of Zigmond and Snaith, and the three-factor models of Friedman, Caci, Brandberg, and Dunbar, it was found that the latent relationships between their dimensions were extremely high (>0.80). This suggests that the dimensions may be overlapping. Therefore, these models were excluded from the following analyses. In the case of Moorey’s model with the correlated factor model, the latent relationship presented a high value (*Φ* = 0.794).

In analyzing the remaining bifactor model (with two orthogonal factors), the explained common variance of the general factor was high (>0.70) and the variances of the specific dimensions were adequate (>0.20). Factorial loads were higher in the general factor than in the specific factors (see Table 3). In addition, the bifactor model presents better goodness-of-fit indices than all previous models. According to the values of the indices to evaluate the bifactor models (*ω*_H_ = 0.80, ECV = 0.72, PUC = 0.54; see Table 3), the existence of one-dimensionality is suggested (Dominguez-Lara and Rodriguez, 2017; Rodriguez et al., 2016). That is why the bifactor model with one general dimension and two specific dimensions of anxiety and depression was selected as the most appropriate. Thus, the rest of the analysis will be carried out with this model.

**Table 3:** Factorial loads and indices of the bifactor model (with two orthogonal factors) of HADS.

	General factor	Anxiety	Depression	R^2^
HADS1	0.628	0.340		0.510
HADS3	0.575	0.503		0.584
HADS5	0.597	0.365		0.490
HADS7	0.651	0.108		0.435
HADS9	0.425	0.252		0.244
HADS11	0.468	0.319		0.321
HADS13	0.531	0.547		0.581
HADS2	0.432		0.878	0.958
HADS4	0.749		0.191	0.597
HADS6	0.738		0.046	0.547
HADS8	0.424		0.165	0.207
HADS10	0.501		0.050	0.254
HADS12	0.714		0.103	0.520
HADS14	0.552		0.182	0.338
Explained common variance (ECV)	0.717	0.309	0.258	-
PUC	0.538	-	-	-
Hierarchical Omega	0.800	0.239	0.113	
Average factorial load (λ_average_)	0.570	0.348	0.231	-

R^2^, Determination coefficient; PUC, Percentage of uncontaminated correlations.

### Measurement invariance

The invariance analysis identified that the bifactor model with one general factor and two specific factors of anxiety and depression presented invariance according to sex and years of education. It was identified that ΔCFI was less than 0.01 in both cases (see Table 4).

**Table 4:** Analysis of factor invariance of the HADS bifactor model (with two orthogonal factors) according to sex and educational years.

	Invariance	Robust X^2^ goodness-of-fit						DIFFTEST		
		Value	df	CFI	RMSEA	SRMR	ΔCFI	Value	df	*p*
Sex	Basal	217.9	126	0.974	0.056	0.051	-	-	-	-
	Metric invariance	238.0	140	0.972	0.055	0.051	−0.002	18.951	14	0.167
	Strong invariance	289.8	165	0.964	0.057	0.058	−0.008	42.908	25	0.014
	Unique factor invariance	310.2	179	0.963	0.056	0.058	−0.002	22.39	14	0.071
Educational years	Basal	299.6	189	0.970	0.062	0.060	-	-	-	-
	Metric invariance	341.8	217	0.966	0.061	0.060	−0.004	39.059	28	0.080
	Strong invariance	403.2	267	0.963	0.057	0.071	−0.003	65.961	50	0.065
	Unique factor invariance	448.5	295	0.958	0.058	0.072	−0.005	40.626	28	0.085

df, degrees of freedom; CFI, comparative fit index; RMSEA, root mean square error of approximation; SRMR, standardized root mean square residual; ΔCFI, variation of the comparative fit index. DIFFTEST, ANOVA difference test.

However, it was found that in the case of sex, the ANOVA test pointed to a significant value (*p* = 0.01) when comparing metric invariance and strong invariance. It was not considered relevant as the *p*-value is very sensitive to sample size.

### Relationship with other variables

The general factor of the HADS showed a moderate correlation with another depression scale (BDI) (*r* = 0.65) and an anxiety scale (BAI) (*r* = 0.67). Additionally, the HADS anxiety-specific factor demonstrated a strong correlation with the Beck Anxiety Inventory (r = 0.70). However, the HADS depression-specific factor showed a moderate correlation with the Beck Depression Inventory (*r* = 0.58; see Table 5), contrary to expectations, as a strong relationship between the two instruments was anticipated.

**Table 5:** Spearman correlation between the dimensions of the HADS with the Beck Depression Inventory (BDI) and the Beck Anxiety Inventory (BAI).

Dimension	HADS—anxiety	HADS—depression	HADS—total
BDI-total	0.59	0.58*	0.65
BAI-total	0.70*	0.50	0.67
M (SD)	7.01 (3.85)	5.88 (3.60)	12.88 (6.69)

All cases have a significant *p*-value (*p* < 0.01). * Main outcomes for the validity of the relationship with other variables.

Additionally, the correlations between the specific anxiety factor and the BAI (*r* = 0.70) were higher than with the BDI (*r* = 0.59), and the correlation between the specific depression factor and the BDI (*r* = 0.58) was higher than with the BAI (*r* = 0.50). Therefore, it is considered that discriminant validity between the different measurements was maintained.

### Internal consistency analysis

The general factor of the HADS showed high internal consistency, with *ω* = 0.91 and *α* = 0.90. Similarly, the specific factors of anxiety (*ω* = 0.84; *α* = 0.84) and depression (*ω* = 0.84; *α* = 0.84) also presented strong consistency coefficients.

## Discussion

### Main findings

The Spanish version of the HADS demonstrates adequate psychometric properties, providing evidence of validity and reliability in an oncological population in Peru. Our findings support the presence of a global factor in the HADS, alongside two specific factors: anxiety and depression. These results align with a meta-analysis based on 21 studies involving 21,820 participants, which found that the bifactorial model with two orthogonal-specific factors exhibited the best psychometric properties (Norton et al., 2013). In our study, the specific depression subdimension showed low factor loadings for most items, which may result in this dimension being underrepresented. Nonetheless, at a general level, the model provides evidence of validity based on internal structure.

Moreover, the HADS can be used to compare both sexes, as well as individuals with varying levels of education. This indicates that the instrument is stable (invariant) across these groups. Additionally, the HADS demonstrates validity about other variables. Specifically, the HADS anxiety subdimension strongly correlates with the BAI, while the global HADS score exhibits moderate correlations with both the BDI and BAI, as hypothesized. However, the correlation between the HADS depression subdimension and the BDI score was moderate. Finally, our study found that the HADS global factor and its specific factors of anxiety and depression demonstrated optimal internal consistency coefficients.

### Factor analysis

Our study identified that the bifactor model is the most appropriate factorial structure of the Spanish version of HADS in cancer patients in Peru. This is in line with what was found in a systematic review performing a meta-confirmation analysis of the HADS, which also concluded that the bifactor model is the most suitable (Norton et al., 2013). Other studies have identified alternative one-, two-, or three-dimensional models (Annunziata et al., 2011; Emons et al., 2010; Gale et al., 2010; Galindo Vázquez et al., 2015a; Matsudaira et al., 2009; Norton et al., 2013; Terol-Cantero et al., 2015). Our study and the meta-confirmation study mentioned above tested these alternative models and agree that the bifactor model is the most adequate.

This could be due to the fact that some HADS studies have used analytical methods that are not suitable or have proven inefficient for psychometric evaluations (e.g., main components, scree plots, eigenvalues, and varimax) (Christensen et al., 2020; Cosco et al., 2012; Gale et al., 2010; Nezlek et al., 2019). Therefore, this could have introduced bias in their measurements, which could have led them to identify the heterogeneity of models. On the other hand, it is worth mentioning that not all studies evaluated the 10 factorial models assessed in our study, so it is possible that other models could potentially have been more appropriate.

The bifactor model consists of a general factor and specific orthogonal factors (where the correlation between factors is zero). In the bifactor model, it is the general factor that strongly explains the variance of the HADS items, and the specific factors explain the variance of a group of items each (depression explains even items; anxiety explains odd items), although these specific factors explain the items less than the general factor. In the HADS, the specific factors identified would be anxiety and depression. As for the general factor, this would be called emotional distress, which is defined as a state of negative affect, suggesting the presence of affective disorders (Vodermaier et al., 2009). We chose to keep the term emotional distress because it is widely used in the literature when referring to the assessment of both anxiety and depression (Lee et al., 2018; Milligan et al., 2018; Yan et al., 2019).

The existence of a general factor that can explain all the items is in line with the proposal of the transdiagnostic models. These models focus on the underlying common symptoms or processes between diagnostic categories (Mansell et al., 2009; Norton and Paulus, 2017). In this study, emotional distress will be the transdiagnostic factor between anxiety and depression present in the HADS. The evidence is not yet conclusive about the single term or transdiagnostic factors present between anxiety and depression. Therefore, we can find in the literature constructs such as dysregulation of negative affect, repetitive negative thinking, and rumination, which are considered transdiagnostic factors for emotional disorders (Akbari et al., 2015; Hofmann et al., 2012; Hsu et al., 2015). On the other hand, the bifactor structure of the HADS appears to solve the problem of overlapping symptoms between anxiety and depression and the high correlation between the factors (anxiety and depression) (Aarstad et al., 2005; Kirkova et al., 2011; Schellekens et al., 2020), stating that both constructs are present in an orthogonal way and it is the general factor that explains most of the variance of the items.

When the HADS was developed, the physical symptoms of anxiety and depression were omitted to avoid confusion with natural physical symptoms associated with patients’ illnesses in hospitals (Zigmond and Snaith, 1983). As a result, the HADS was originally designed to assess the emotional and cognitive aspects of anxiety and depression. Transdiagnostic models do not contradict the presence of specific factors such as anxiety and depression, as they do not pretend to oppose specific diagnoses. Instead, they suggest using specific models or transdiagnostic depending on whether it is clinically significant or whether the presence of specific diagnoses is necessary, which may well complement the information provided by the transdiagnostic factors (Mansell et al., 2009). Finally, although specific anxiety and depression factors are identified in the structure, it is advisable to exercise caution in considering both dimensions as sufficient for diagnosing anxiety and depression. This would require further evaluation.

In terms of usefulness, three strengths were identified in the HADS bifactor structure. First, the HADS would be a versatile instrument, which would work very well as a filter to identify emotional distress (transdiagnostic factor) and would allow specifying the specific symptomatology (i.e., presence of depression or anxiety symptoms). This would be very useful in terms of further evaluation, giving more information about whether the patient has any emotional disorder and whether it is more specifically anxiety and/or depression. Second, it is important to note the brevity of the HADS, with the 14 items, it has proven to be of great value in detecting emotional distress and symptoms of anxiety and depression. Third, the HADS is a tool that stands out for its configuration, in which physical symptoms are not considered. This is noteworthy because it decreases the likelihood of false positives due to the physical symptoms experienced by hospital patients, which are often confused with the physical symptoms of anxiety and depression.

### Measurement invariance

A crucial aspect of clinical assessment is to determine whether the instrument is invariant between different groups, i.e., whether the two or more groups can understand the construct equivalently assessed by the scale and thus make comparisons between those groups (Putnick and Bornstein, 2016). As if an instrument is not invariant among different groups, different sensitivity and specificity analyses will be required, which would limit its clinical use, to name one example.

Our study found that there are no differences in the factor structure of the HADS in the Peruvian oncological population based on sex, as previously evidenced in other studies conducted in a sample of the Italian community and HIV patients in China (Iani et al., 2014; Yang et al., 2019). This suggests that the HADS is useful for detecting symptoms of emotional distress, anxiety, and depression in both sexes, although the clinical manifestations of depression and anxiety may vary according to sex (Zarragoitía Alonso, 2013). It is important to note that other HADS studies, which proposed alternative two-dimensional or three-dimensional factor structures, have also found evidence of invariance between men and women (Annunziata et al., 2011; Czerwiński et al., 2020; Fong and Ho, 2014; Hunt-Shanks et al., 2010; Stott et al., 2017a; Stott et al., 2017b). This is an encouraging finding as the instrument appears to allow for the assessment of anxiety, depression, and emotional distress without distinction by sex in different populations, even when less adequate factorial models are used.

On the other hand, it was shown that the HADS can measure the symptoms of emotional distress, anxiety, and depression in the Peruvian cancer population with different years of study (less than 6 years, between 7 and 11 years, and 12 or more years), one relevant point is that our study has been the first to evaluate the measurement invariance of the HADS based on educational level. It had previously been pointed out that the uneven distribution of the elements of inverse writing could influence vulnerable populations such as individuals with low levels of education due to the difficulty that it would generate in reading activity (Lin et al., 2017). However, these results support that the instrument has an equal factorial structure, the items contribute similarly, and the intersections are equivalent in the groups. Despite the heterogeneous characteristics of the sample, which comes from an institute specialized in cancer and includes populations from different geographical areas of Peru and varying levels of education, this allows us to affirm that the variables are evaluated in the same way across all groups. This is a valuable feature for making informed public health decisions.

### Relationship with other variables

Our study found that the specific depression factor of the HADS correlates more strongly with another instrument measuring depression (BDI) than with an instrument measuring anxiety (BAI). Similarly, the specific anxiety factor of the HADS shows a stronger correlation with the BAI than with the BDI. This suggests that the HADS provides evidence of convergent validity, as dimensions measuring the same construct exhibit stronger relationships than those measuring different constructs (Muñiz, 2018). Our findings are supported by previous studies that identified similar relationships in patients with chronic illnesses (Preljevic et al., 2012; Schellekens et al., 2016).

### Internal consistency analysis

In the bifactorial model of the HADS were found acceptable levels of reliability (*ω* > 0.70 and *α* > 0.70) for both the general factor and the specific factors, which coincide with the results of other studies (Cabrera et al., 2015; Galindo Vázquez et al., 2015b; Li et al., 2016; Martínez López et al., 2012; Terol-Cantero et al., 2015). Having an acceptable level of reliability strengthens and enhances the relevance of using the HADS, as it demonstrates that the instrument provides a good degree of stability in its measurements.

### Relevance in public health and psychosocial providers

This study provides different evidence of the validity and reliability of the HADS in the Peruvian oncological population, which supports its use within the context of oncological patient care. The HADS can be used as a tool to evaluate the clinical progress of individuals receiving psychological care in an oncological context. In addition, it can be used as a research tool in clinical trials or longitudinal studies in cancer patients, as it is an instrument with solid evidence of validity and reliability.

The Peruvian health system is overburdened, and mental health professionals are insufficient and have very little time to treat patients (Toyama et al., 2017). Thus, the HADS, because of its brevity (only 14 items) and empirical support, could be a good option for assessing depressive and anxiety symptoms within the hospital setting. Mainly in rural areas of Peru, where the percentage of mental healthcare is much lower (Villarreal-Zegarra et al., 2020) Our findings may be of interest to public health, as implementing the HADS within the primary or hospital care system would help streamline and expedite the processes of identifying and referring patients with any of these symptoms. Therefore, we encourage policymakers to consider incorporating the HADS into clinical practice guidelines.

### Strengths and limitations

One of the strengths of our study is the certainty that the participants had cancer as they all had previous medical examinations to confirm the disease. However, our study has five major limitations. First, it does not provide a cohort point for identifying whether participants have symptoms of depression, anxiety, or emotional distress. Therefore, future studies on sensitivity and specificity are needed. Second, our data were selected in a non-probabilistic way, so our results cannot be generalized to the entire cancer population in Peru. Third, because we had a small sample size, we could not perform analyses of variance among other interest groups such as age, income, living in rural and urban areas, or stages of cancer. For example, the necessary assumptions to perform a measurement invariance analysis between age groups were not met, which caused the models to fail to converge. Fourth, the relationship of the HADS with other clinically relevant variables such as quality of life, wellbeing, or other instruments of emotional distress could not be assessed (Mansell et al., 2009; Milligan et al., 2018; Norton and Paulus, 2017). Fifth, our study was non-probabilistic, so the findings could not be generalized to other populations.

## Conclusion

Our results support the use of the HADS in the oncological population in Peru, as it demonstrates evidence of both validity and reliability. Our data support a bifactor model of the HADS, with one general factor of emotional distress and two specific factors (anxiety and depression). In addition, it is invariant, presents convergent validity, and has adequate internal consistency coefficients.

## Data Availability

The datasets presented in this study can be found in online repositories. The names of the repository/repositories and accession number(s) can be found at: the data used in the analysis is attached as https://doi.org/10.6084/m9.figshare.13626773.v2.
